# Complete Genome Sequence of *Bacillus velezensis* YJ11-1-4, a Strain with Broad-Spectrum Antimicrobial Activity, Isolated from Traditional Korean Fermented Soybean Paste

**DOI:** 10.1128/genomeA.01352-17

**Published:** 2017-11-30

**Authors:** Hyo Jung Lee, Byung-Hee Chun, Hye Hee Jeon, Yeon Bee Kim, Se Hee Lee

**Affiliations:** a Department of Biology, Kunsan National University, Gunsan, Republic of Korea; b Department of Life Science, Chung-Ang University, Seoul, Republic of Korea; c Microbiology and Functionality Research Group, World Institute of Kimchi, Gwangju, Republic of Korea

## Abstract

*Bacillus velezensis* YJ11-1-4 is a strain that exhibits broad-spectrum antimicrobial activity against various pathogens. It was isolated from doenjang, a traditional Korean fermented soybean paste. The genome comprises a single circular chromosome of 4,006,637 bp with 46.42% G+C content without plasmids.

## GENOME ANNOUNCEMENT

*Bacillus velezensis* was first proposed by Ruiz-García et al. (1) as a surfactant-producing bacterium and was subsequently validated in a later heterotypic synonym of *B. amyloliquefaciens* based on DNA-DNA hybridization (2). Recently, Dunlap et al. (3) suggested that *Bacillus methylotrophicus*, *Bacillus amyloliquefaciens* subsp. *plantarum*, and “*Bacillus oryzicola*,” reported as plant pathogen antagonists, should be reclassified as a heterotypic synonym of *B. velezensis* based on phylogenomic analysis using core genomes. Because diverse foodborne pathogens have been detected from doenjang samples, the use of a starter culture with a broad-spectrum antimicrobial activity is important for food safety during fermentation. Therefore, *Bacillus velezensis* YJ11-1-4 (Korean Agricultural Culture Collection [KACC] 18396) which has revealed good antimicrobial activities against various foodborne pathogens, including *Bacillus cereus*, *Escherichia coli*, *Listeria monocytogenes*, *Staphylococcus aureus*, and *Aspergillus flavus* subsp. *Flavus*, was isolated from homemade doenjang, and its complete genome was sequenced.

The whole genome of *B*. *velezensis* YJ11-1-4 was sequenced using a Pacific Biosciences RS II platform with a 20-kb SMRTbell template library at Macrogen (Seoul, Republic of Korea). It generated a total of 969,934,944 bp (about 198-fold coverage) with 112,837 reads. The sequencing reads were assembled using the Hierarchical Genome Assembly Process version 2.0 (HGAP2.0). The assembled genome of strain YJ11-1-4 comprises a single circular chromosome of 4,006,637 bp with a 46.42% G+C content without plasmids. The complete genome sequence was annotated using the NCBI Prokaryotic Genomes Automatic Annotation Pipeline (PGAAP) (http://www.ncbi.nlm.nih.gov/genomes/static/Pipeline.html). The tRNA and rRNA genes were detected using tRNAscan-SE (4) and RNAmmer version 1.2 (5) software, respectively. Strain YJ11-1-4 contains 3,586 predicted protein-coding sequences. The chromosome harbors 86 tRNA genes for 20 amino acids and 9 rRNA operons.

Genomic analysis reveals that the genome contains two complete antibiotic biosynthesis operons of bacilysin (targeting a wide range of bacteria and the yeast *Candida albicans*) encoded by *bacABCDE* (AAV30_01545 to AAV30_01565) (6) and amylocyclicin (targeting mostly Gram-positive bacteria) encoded by *acnABCDEF* (AAV30_04380 to AAV30_04405) (7). Strain YJ11-1-4 harbors many operons encoding nonribosomal synthesized antimicrobial peptides, including fengycin (targeting fungi) encoded by *fenEDCBA* (AAV30_10135 to AAV30_10160), bacillomycin D (targeting yeast) encoded by *bmyDABC* (AAV30_10275 to AAV30_10290), surfactin (targeting virus) encoded by *srfABCD* (AAV30_17390 to AAV30_17405), and an LCI gene (targeting some Gram-negative bacteria) (AAV30_17550) (6). Several biosynthesis clusters of antibacterial polyketides, such as bacillaene (AAV30_10840 to AAV30_10905), macrolactin (AAV30_12135 to AAV30_12175), and difficidin (AAV30_07950 to AAV30_08020), were also found in the genome. In conclusion, genomic analysis suggests that *B. velezensis* YJ11-1-4 can be used as a biocontrol agent of various foodborne pathogens in doenjang.

### Accession number(s).

The genome information for *B. velezensis* YJ11-1-4 was deposited in NCBI under GenBank accession number CP011347.
